# Supplementary material for: Structural Role of Plasma Membrane Sterols in Osmotic Stress Tolerance of Yeast Saccharomyces cerevisiae
Source: Membranes (Basel). 2022 Dec 17;12(12):1278. doi: 10.3390/membranes12121278 (PMC9781751; doi:10.3390/membranes12121278)
Supplement: Supplementary file 1 [file membranes-12-01278-s001.zip › membranes-2024551-supplementary.pdf]

Supplementary Materials

Structural Role of Plasma Membrane Sterols in Osmotic Stress Tolerance of Yeast *Saccharomyces cerevisiae*

Svyatoslav S. Sokolov, Marina M. Popova, Peter Pohl, Andreas Horner, Sergey A. Akimov, Natalia A. Kireeva, Dmitry A. Knorre, Oleg V. Batishchev, Fedor F. Severin

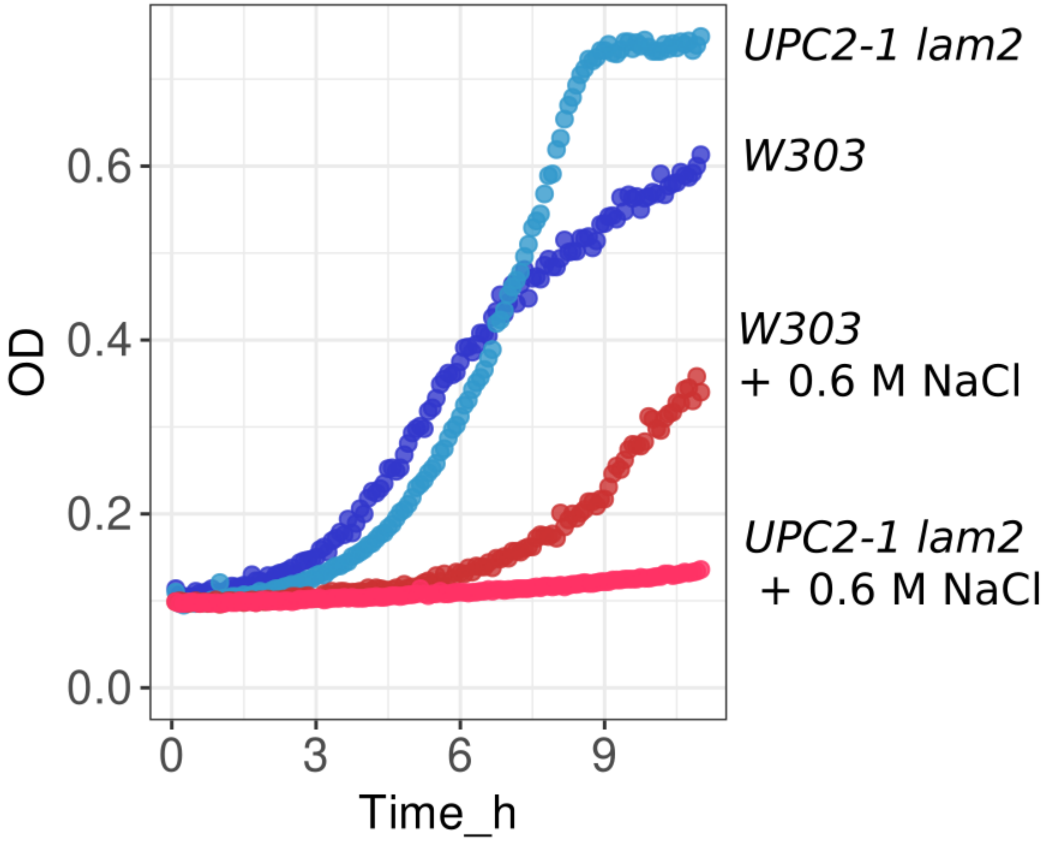

Figure S1. Typical growth curves of yeast cells in YPD and in YPD containing 0.6M NaCl.

Table S1. Growth rates of a set of mutants in absence and in the presence of the osmolytes.

	**parentalstrain**	**control**	**0.6 M NaCl**	**0.6 M KCl**	**1.2M sorbitol**
BY4741	*BY4741*	0.36 ± 0.01	0.27 ± 0.03	0.3 ± 0.02	0.28 ± 0.02
Δhog1	*BY4741*	0.38 ± 0.01	0.16 ± 0.04	0.2 ± 0.01	0.09 ± 0.03
Δerg2	*BY4741*	0.32 ± 0.02	0.25 ± 0.01	0.28 ± 0.01	0.27 ± 0.02
Δerg3	*BY4741*	0.39 ± 0.02	0.31 ± 0.04	0.39 ± 0.01	0.31 ± 0.01
Δerg4	*BY4741*	0.33 ± 0.02	0.21 ± 0.05	0.22 ± 0.01	0.25 ± 0.04
Δlam2	*BY4741*	0.35 ± 0.02	0.28 ± 0.02	0.29 ± 0.02	0.29 ± 0.03
Δerg2Δlam2	*BY4741*	0.36 ± 0.03	0.24 ± 0.05	0.32 ± 0.02	0.22 ± 0.01
Δerg3Δlam2	*BY4741*	0.39 ± 0.02	0.33 ± 0.02	0.37 ± 0.02	0.28 ± 0.05
Δerg4Δlam2	*BY4741*	0.3 ± 0.02	0.06 ± 0.06	0.18 ± 0.03	0.23 ± 0.03
W303	*W303*	0.31 ± 0.06	0.27 ± 0.08	0.3 ± 0.08	0.21 ± 0.05
Δlam1Δlam2Δlam3Δlam4Δosh3	*W303*	0.28 ± 0.07	0.26 ± 0.07	0.28 ± 0.05	0.21 ± 0.08
Δlam1Δlam2Δlam3Δlam4Δosh4Δosh5	*W303*	0.21 ± 0.05	0.17 ± 0.06	0.2 ± 0.03	0.17 ± 0.05
Δlam1Δlam2Δlam3Δlam4	*W303*	0.26 ± 0.05	0.22 ± 0.09	0.24 ± 0.09	0.2 ± 0.07
Δlam5Δlam6	*W303*	0.35 ± 0.08	0.24 ± 0.07	0.28 ± 0.06	0.19 ± 0.07
Δlam1Δlam2Δlam3Δlam4Δlam5Δlam6	*W303*	0.32 ± 0.06	0.27 ± 0.06	0.26 ± 0.02	0.2 ± 0.07
UPC2-1	*W303*	0.31 ± 0.04	0.16 ± 0.06	0.32 ± 0.08	0.34 ± 0.05
UPC2-1Δlam2	*W303*	0.28 ± 0.07	0.06 ± 0.06	0.23 ± 0.03	0.2 ± 0.02
Δlam1Δlam2Δlam3Δlam4osh1Δosh2	*W303*	0.32 ± 0.08	0.27 ± 0.05	0.29 ± 0.07	0.19 ± 0.05
Δerg3	*W303*	0.33 ± 0.08	0.26 ± 0.06	0.3 ± 0.08	0.23 ± 0.05
Δlam1Δlam2Δlam3Δlam4Δerg3	*W303*	0.31 ± 0.07	0.26 ± 0.06	0.28 ± 0.06	0.2 ± 0.08
